# Improved self-efficacy in persons with relapsing remitting multiple sclerosis after an intensive social cognitive wellness program with participation of support partners: a 6-months observational study

**DOI:** 10.1186/1477-7525-12-40

**Published:** 2014-03-19

**Authors:** Peter Joseph Jongen, Rob Ruimschotel, Marco Heerings, Astrid Hussaarts, Lotte Duyverman, Anneke van der Zande, Joyce Valkenburg-Vissers, Hanne Wolper, Maarten van Droffelaar, Wim Lemmens, Rogier Donders, Leo H Visser

**Affiliations:** 1 MS4 Research Institute, Ubbergseweg 34, 6522 KJ Nijmegen, The Netherlands; 2 Medical Psychiatric Centre PsyToBe, Metroweg 50, 3083 BB Rotterdam, The Netherlands; 3 National Multiple Sclerosis Foundation, Wagenstraat 25, 3142 CR, Maassluis, The Netherlands; 4 Body-Care Verbeek, Ekkersrijt 4206, 5692 DE Son, The Netherlands; 5 Department for Health Evidence, Radboud University Nijmegen Medical Centre, P.O. Box 9101, 6500 HB Nijmegen, The Netherlands; 6 St. Elisabeth Hospital, Hilvarenbeekseweg 60, 5022 GC Tilburg, The Netherlands; 7 University of Humanistic Studies, Kromme Nieuwegracht 29, 3512 HD Utrecht, The Netherlands

**Keywords:** Self-efficacy, Multiple sclerosis, Autonomy, Participation, Quality of life, Relapsing remitting, Progressive, Depression, Anxiety, Fatigue

## Abstract

**Background:**

For persons with multiple sclerosis (MS) it is important to preserve their autonomy, in spite of increasing disability. A major factor mediating autonomy is self-efficacy. According to the social cognitive theory stressors are crucial determinants of self-efficacy, as well as the interaction with partners.

**Methods:**

In an explorative observational study we assessed in 47 persons with MS (PwMS) the effect of an intense, multidisciplinary, 3-day, social cognitive wellness program with the participation of support partners, after 1, 3 and 6 months. Primary outcomes: self-efficacy-control and -function (Multiple Sclerosis Self-Efficacy Scale [MSSES]),limitations to and problems with participation and autonomy (Impact on Participation and Autonomy [IPA] scale). Secondary outcomes: health-related quality of life (HRQoL) (MS Quality of Life-54 Items [MSQoL-54] questionnaire), anxiety, depression (Hospital Anxiety and Depression Scale [HADS]), and fatigue (Modified Fatigue Impact Scale-5 Items [MFIS-5]). Disability was measured with the Expanded Disability Status Scale (EDSS). Percentage changes from baseline were tested with T-tests, level of significance 0.05.

**Results:**

In the whole group the MSQoL-54 Mental score was increased at 1, 3 and 6 months (+16.0%, +13.2%, +12.2%), and the MSQoL-54 Physical (+10.2%) at 6 months, with no changes in other outcomes. The relapsing remitting (RR) subgroup (n = 20) had at 6 months an increase in the MSSES-Control score (+24.8%) and in the MSQoL54 Mental and Physical scores (+22.3%, +17.6%). Progressive patients (n = 22) only showed an increase in the MSQoL-54 Mental score (+11.5%) at 1 month. In the low-disability (EDSS < 4.0) subgroup the MSSES-Control score was increased (+23.8%) at 6 months, and the IPA-Limitations and -Problems scores decreased at 3 months (−6.1%, −8.8%); the MSQoL-54 Mental score had increased at 1, 3 and 6 months (+19.3%, +21.5%, +19.3%). In the high-disability (EDSS > =4.0) subgroup no significant changes occurred.

**Conclusions:**

Results from this observational study suggest that 6 months after an intense, 3-day, multidisciplinary, social cognitive wellness program with support partners, PwMS with a RR course or low disability may experience an improved self-efficacy-control and HRQoL.

## Background

Multiple sclerosis (MS) is a chronic inflammatory and degenerative disease of the central nervous system (CNS). In about 80% of the persons with MS (PwMS) the initial phase is clinically characterized by relapses followed by a complete or partial recovery: relapsing remitting MS (RRMS). Although disease modifying drugs (DMDs) decrease the frequency and severity of relapses, the disease course remains largely unpredictable. After 10 to 15 years RRMS frequently progresses to secondary progressive MS (SPMS), that is characterized by a slow unceasing increase in disability. It is uncertain to what extent DMD treatment of RRMS may prevent or postpone SPMS. About 15% of PwMS have a progressive course from disease onset: primary progressive MS (PPMS). In both SPMS and PPMS DMDs are not effective.

Most PwMS get increasingly disabled in the course of the disease, with disabilities negatively affecting people’s independence. Moreover, the negative experience of losing independence has a negative impact on self-efficacy. Self-efficacy is a psychological concept that refers to the degree in which a person is confident to complete tasks and reach goals in specific situations [1]. It is a core component in social cognitive theory, in which psychosocial functioning is determined by reciprocal interactions between personal factors, behavior, and the environment [2,3]. Low self-efficacy has been associated with lower health-related quality of life (HRQoL) [4,5], less psychological adjustment [6], and less physical activity [5]. Since a lowered self-efficacy causes PwMS to undervalue their actual capabilities, a vicious circle may start and still existing functions run a risk to eventually disappear.

Partners and other informal caregivers, like household and family members, friends and neighbors, have also to deal with the consequences of worsening disabilities and a diminishing self-efficacy in PwMS. A constant and growing appeal by PwMS on their support partners results in an increasing mental and physical strain of the latter, and may seriously weigh on the mutual relation.

A wellness program is a structured intervention focused on achieving wellness in the physical, psychological and spiritual realm [7]. In the U.S.A., Can Do MS, a publically accessible nonprofit wellness program, offers concentrated 4-day interdisciplinary educational wellness programs for PwMS, that promote health seeking behaviors, lifestyle empowerment, and wellness including exercise, and with optional participation of support partners [8]. This educational Can Do MS Program (CDP) has been developed to enable PwMS the uncovering of their existing capabilities [8]. Recently Ng et al. observed an improvement in self-efficacy and perceived health after CDP [8]. Although a complementary program for participants’ support partners was an integral part of this CDP, it is unclear whether such participation was mandatory and if not, to what extent support partners did participate in the program.

In view of the positive results of the educational CDP reported by Ng et al., the central role of self-efficacy in social cognitive theory, and the importance of optimal relations between PwMS and their support partners, we set ourselves to assess the effect of a Social Cognitive Can Do program (SCDP) with the participation of support partners. Our primary intention was to exploratively investigate whether changes in self-efficacy, autonomy and participation could be observed after SCDP (proof of concept) and at what time points eventual changes were most prominent. Secondarily, we assessed changes in HRQoL, anxiety, depression, and fatigue, and also performed separate analyses for persons with RRMS, progressive MS, low disability (Expanded Disability Status Scale [EDSS] < 4.0) and high disability (EDSS > =4.0).

## Methods

### Social cognitive can do program

#### Concept

The goal of the SCDP is to uncover and promote existing capabilities, with the notion ‘stressor’ as central concept. It is primarily a sociologically oriented approach, as it tries to identify stressors that confine PwMS to their physical, psychological or social roles. To reduce these stressors, SCDP is based on the following principles: identification and reduction of existing stressors; client-centeredness; inclusion of the partner or another significant informal caregiver; group sessions; and self-reliance, autonomy, and acceptance as central themes. Accordingly, SCDP focuses on the exploration of stressors that confine PwMS to their disease and their limitations; reduces relevant stressors; explores and pushes personal boundaries; and creates new personal boundaries by making optimal use of the existing potential. To place the individual’s capabilities in a realistic framework SCDP’s central mottos are ‘Can’, ‘Will’, ‘Choose’, ‘Open up to others’, and ‘Do’. SCDP’s message is that by exploring their boundaries PwMS become more aware of their faculties, and that the resulting self-management leads to higher awareness of potentials and a better communication with care professionals.

#### Components

SCDP’s components are 1) large group sessions, 2) small group sessions, 3) consultations (carrousel), 4) a theatre evening, and 5) start of the day with a joint activity (optionally).

##### Large group sessions

Plenary sessions: participants make optimal use of their existing potentials, learn how to support and encourage other participants, and experiment how to give the required feedback to the multidisciplinary team. Group sessions in which half the participants take part: participants examine and identify which stressors have to be addressed most, and formulate realizable individual aims (one or two).

##### Small group sessions

The small group sessions form the actual training. Depending on their individual goals the participants sign up for the training groups ‘Body’, ‘Feeling’ or ‘Life’, to work out their aims and to experiment whether they can reduce their stressors. The Body sessions focus on the exploration of the physical capabilities and are coached by a physiotherapist. The Feeling sessions focus on the exploration of the emotional potential and are coached by a psychiatrist and a psychiatric nurse. The Life sessions focus on the exploration of capabilities relating to the daily living with MS and are coached by a neurologist, a registered nurse specialized in MS, and a person with MS. In addition, there are relaxation sessions for those who have difficulties in experiencing their body: the Yoga session focuses on body experience and relaxation, whereas the Physical session focuses on relaxation through physical strain. The choices between the various small group sessions are made independently by the participants.

##### Consultations

After having identified and formulated in the large group sessions their individual stressors and aims, the participants sign in for one or more group consultations, during which they verify whether their aims are realizable by asking the members of the multidisciplinary team for aim-related medical information.

##### Theater evening

On the informal theater evening the participants practice to change roles and show their potentials by openly experimenting. They do their best to perform before each other and the team. The jointly created evening performance increases the cohesion within the group and learns participants to find an equilibrium between consuming and action.

##### Joint activity at the start of the day

During an optional joint activity (walk in the woods) at the start of the day the participants experiment with physical challenges and with the management of their energy.

#### Multidisciplinary team

The multidisciplinary team includes a psychiatrist, psychiatric nurse, neurologist, specialized MS nurse, physiotherapist, yoga teacher, and a person with MS. The team members respect and understand the participants’ individual qualities and differences, and they stimulate, defy and confront them to explore and push their boundaries. Apart from the consultations the team keeps to coaching, stimulating and activating the participants. By participating in all large group sessions the team members become acquainted with the individual stressors and goals. During the consultations they have a professional and informative role. In the small group sessions every discipline focuses on its own area of interest. During a tip time at the end of each day the team members evaluate the sessions, inform each other on the participants’ progresses and obstacles, discuss whether the participants make optimal use of their opportunities, and monitor to what extent the personal goals are being attained.

### Study design and organization

The SCDP was given during three days in Zorghotel Spelderholt, Beekbergen, the Netherlands, a facility especially equipped for the accommodation of people with impaired health. From March 2012 to December 2012 five weekends were organized for PwMS and their significant support partner (partner or informal caregiver), i.e. 8 to 10 couples per weekend. The SCDP was given from Friday to Sunday.

The idea for a social cognitive intervention with partner or other informal care giver (significant other) was conceived by the National Multiple Sclerosis Foundation (NMSF), Maassluis, the Netherlands (AvdZ) and further developed in collaboration with PsyToBe (RR) and the other team members. The weekends were organized and managed by the NMSF, Maassluis, the Netherlands (MvD). The observational study was conducted by the MS4 Research lnstitute, Nijmegen, the Netherlands (PJJ, MH).

PwMS and their support partners were recruited through (a) announcements on the NMSF’s website http://www.nationaalmsfonds.nl, (b) advertisements in ‘Nieuwslijn’, the NMSF’s quarterly journal and (c) email messages to all registered members of the NMSF. Those interested in participation contacted the NMSF by telephone. They were informed on the study and its procedures, had their questions answered, and underwent a screening interview and inclusion procedure. The inclusion criteria were (a) definite MS, (b) relapse free in the last 4 weeks, and (c) able and willing to participate in the SCDP and the study-related assessments.

The study used paper-and-pencil self-report questionnaires that were sent by regular mail one week before the weekend, and 1, 3 and 6 months after the intervention. The questionnaires were accompanied by a stamped return envelope addressed to the MS4 Research Institute.

### Outcomes and outcome measures

Self-efficacy and participation/autonomy were the primary study outcomes. HRQoL, anxiety, depression, and fatigue were secondary outcomes.

#### Primary outcome measures

Self-efficacy was assessed by the Multiple Sclerosis Self-Efficacy Scale (MSSES). The MSSES is an 18-item, psychometrically validated, self-report questionnaire for the assessment of self-efficacy [9]. The MSSES consists of two 9-item subscales of Function and Control. Each item is scored on a Likert-like scale form 10 (very uncertain) to 100 (very certain) and addition of the respective item scores yields the MSSES-Function score and the MSSES-Control score, both ranging from 100 (minimum) to 1000 (maximum). The MSSES-Function subscale measures confidence with functional abilities, whereas the MSSES-Control subscale measures confidence with managing symptoms and coping with the demands of illness [9].

The Impact on Participation and Autonomy (IPA) questionnaire is a 32-item, psychometrically, validated, generic, self-report instrument for the quantification of limitations in participation and autonomy in people with chronic health conditions [10,11]. The IPA-Limitations subscale assesses perceived limitations in participation and autonomy in relation to 32 different life situations across five subscales: autonomy indoors, family role, autonomy outdoors, social life and relationships, and work and education [10-12]. Items are rated on a 5-point scale from 0 (very good) to 4 (very poor), and a higher score indicates a higher limitation to participation and autonomy. The IPA-Problems subscale examines the extent to which these limitations are experienced as problematic, by assessing nine different areas of participation and autonomy: mobility, self care, activities in and around the house, looking after money, leisure, social life and relationships, paid or voluntary work, education and training, and helping and supporting other people [10-12]. The perceived problems are graded on 3-point scale ranging from 0 (no problem) to 2 (severe problems), and a higher IPA-Problems score indicates a greater experience of problems [10-12].

#### Secondary outcome measures

HRQoL was assessed by the Multiple Sclerosis Quality of Life 54-Item (MSQoL-54) questionnaire [13]. The MSQoL-54 is a psychometrically validated, MS-specific, multi-dimensional inventory of patient-centered health status, and consists of the Short Form 36-Item (SF-36) health survey as a generic core measure, supplemented with 18 questions on items relevant to PwMS in the areas of health distress, sexual function, satisfaction with sexual function, overall quality of life, cognitive function, energy, and pain and social function [13]. The MSQoL-54 contains 52 items distributed into 12 scales, and two single items. A physical and a mental dimension underlie the MSQoL-54: the Physical and Mental domains [13]. Scores for each domain range from 0 to 100, where higher values indicate better HRQoL.

Anxiety and depression were measured by the Hospital Anxiety and Depression Scale (HADS), a psychometrically validated, 14-item, self-report questionnaire for anxiety and depression [14]. The HADS consists of two subscales, one for anxiety and one for depression, each comprising seven questions. Each question scores 0 to 3 points, and a total subscale score of 0 to 7 points indicate no anxiety/depression, 8 to 10 points indicate possible mild to moderate symptoms of anxiety/depression, and 11 to 21 points indicate a probable clinically significant condition of anxiety/depression [14].

Fatigue was measured by the Multiple Sclerosis Fatigue Impact Scale 5-Item Version (MFIS-5), a validated, short questionnaire examining a patient’s perceived impact of fatigue on a variety of daily activities over the past month [15]. Answers to each question are rated on a 5-point scale from 0 to 4. The MFIS-5 total score consists of the sum of the raw scores on these 5 items and ranges from 0 to 20, where higher scores indicate more experienced fatigue [15].

Disability was measured by an assessment of the Expanded Disability Status Scale (EDSS) score via telephone [16]. The classical EDSS is based on a neurological examination that provides the basis for the assessment of several functional systems that, according to predefined algorithms, contribute to the EDSS score [17]. An EDSS version for use by telephone via a structured interview has been developed and validated [16].

### Ethical aspects

The study protocol was submitted to and viewed by the ethical review board “Medisch-Ethische Toetsing Onderzoek Patiënten” (METOPP), Tilburg, the Netherlands. CCMO (Central Committee on Research Involving Human Subjects) number: NL36051.028.11 (http://www.ccmo.nl/en). The METOPP concluded that because of the observational design of the study a formal review by an ethical review board was not required, as the study did not meet the criteria stated in the Dutch Medical Research Involving Human Subjects Act of 1999. The study was carried out in compliance with the Declaration of Helsinki. Potential participants were informed on the study by the NMSF and two members of the multidisciplinary team (AH, PJJ). Patients who agreed to participate signed an informed consent form. The SCDP constitutes an unusual mental and physical pressure and might therefore lead to the temporary occurrence or worsening of MS symptoms, like fatigue, mood alteration, or emotions. The continuous presence of the experienced team guaranteed that unwanted changes were rapidly noticed and adequately cared for.

### Data analysis

For both primary and secondary outcomes the absolute values at baseline and at 1, 3 and 6 months after SCDP are described as mean with standard deviation (SD), and the percentage changes from baseline at 1, 3 and 6 months are described as mean with standard error of the mean (SEM). As our intention was to explore whether any changes could be observed after SCDP (proof of concept) and at what time points eventual changes were most prominent, we choose to compare each outcome at each time point with its baseline, by using multiple paired Student T-tests. To better assess the degree and clinical relevance of changes we tested for each outcome at each time point the percentage change compared to baseline instead of absolute values. Similar analyses were performed in subgroups according to disease course (RR, progressive) and degree of disability (EDSS < 4.0, EDSS > = 4.0). Differences in demographic and disease characteristics between RR and progressive patients were tested with unpaired Student T-tests. For all tests P-values <0.05 were considered significant.

## Results

### Participants

Ninety-four participants, 47 PwMS and their support partners, were included in the study and participated in a SCDP. Eighty-seven (92.6%) persons completed the program, whereas seven persons (7.4%), being three couples and one support partner, stopped prematurely. Reasons for couples to discontinue were: knee symptoms in a person with MS (1×), death of a close relative (1×), and refusal of a partner to comply with the program (1×); one partner left prematurely due to an exacerbation of pre-existing marital problems. The drop outs occurred in the 1st (n = 2), the 3rd (n = 3) and the 4th (n = 2) SCDP weekend. In five persons (5.1%) the discontinuation was considered SCDP-related.

Follow up data were obtained from 44 PwMS. Twenty had a RR, 22 a SP, and two a PP disease course. The demographic and disease characteristics are presented in Table 1. As expected, the mean values for age, disease duration and EDSS score were significantly lower in the RR group than in the progressive group (all P values <0.05).

**Table 1:** Mean (standard deviation) (minimum-maximum) values of demographic and disease characteristics of PwMS participating in SCDP

	**Relapsing remitting (n = 20)**	Progressive (n = 24)*
Male/female ratio	4/16 (1:4)	5/19 (1:3.8)
Age (yrs.)**	42.7 (10.1) (25–65)	48.7 (7.6) (30–60)
Disease duration (yrs.)**	8.4 (6.9) (1.2-24.2)	17.5 (8.6) (3.2-36.0)
EDSS score**	3.1 (1.2) (1.5-6.0)	5.5 (1.4) (3.0-7.5)

*, Secondary Progressive (n = 22) and Primary Progressive (n = 2); **, P < 0.05 for comparisons between Relapsing Remitting and Progressive persons.

### Self-efficacy, participation and autonomy

The mean (SD) MSSES-Function, MSSES-Control, IPA-Limitations and IPA-Problems values for the total group at baseline and at 1, 3 and 6 months are presented in Table 2. The mean (SEM) percentage changes from baseline at 1, 3 and 6 months in the total group are presented in Table 3. Not a single change reached statistical significance.

**Table 2:** Mean (SD) values of Multiple Sclerosis Self-Efficacy Scale (MSSES) and Impact on Participation and Autonomy (IPA) scores at 1, 3 and 6 months after SCDP in the total group and in the Relapsing Remitting (RR) and Progressive (P) subgroups

	**Baseline**			**1 month**			**3 months**			**6 months**		
	**Total**	**RR**	**P**	**Total**	**RR**	**P**	**Total**	**RR**	**P**	**Total**	**RR**	**P**
MSSES-Function	66.45 (21.52)	81.80 (12.15)	53.59 (19.62)	66.28 (21.29)	81.70 (12.52)	54.09 (19.31)	64.47 (22.41)	74.75 (19.33)	54.23 (20.28)	66.10 (21.93)	81.30 (12.00)	50.94 (18.07)
MSSES-Control	47.79 (19.06)	57.01 (19.67)	41.63 (15.29)	49.33 (17.42)	58.04 (15.01)	43.84 (16.24)	49.64 (19.76)	53.52 (18.28)	45.50 (20.77)	55.05 (18.91)	64.26 (13.66)	46.18 (19.44)
IPA-Limitations	2.70 (0.57)	2.43 (0.57)	2.89 (0.50)	2.59 (0.63)	2.34 (0.53)	2.76 (0.65)	2.57 (0.62)	2.26 (0.51)	2.85 (0.56)	2.58 (0.67)	2.30 (0.55)	2.88 (0.64)
IPA-Problems	2.09 (0.36)	1.95 (0.36)	2.17 (0.32)	1.97 (0.46)	1.73 (0.39)	2.11 (0.43)	1.93 (0.52)	1.74 (0.48)	2.07 (0.54)	2.02 (0.51)	1.86 (0.52)	2.14 (0.48)

**Table 3:** Mean (SEM) percentage changes from baseline in Multiple Sclerosis Self-Efficacy Scale (MSSES) and Impact on Participation and Autonomy (IPA) scores at 1, 3 and 6 months after SCDP in the total group and in the Relapsing Remitting (RR) and Progressive (P) subgroups

	**1 month**			**3 months**			**6 months**		
	**Total**	**RR**	**SP/PP**	**Total**	**RR**	**SP/PP**	**Total**	**RR**	**SP/PP**
MSSES-Function	4.5% (4.1)	0.9% (10.0)	15.1% (47.2)	−0.2% (5.2)	6.2% (21.9)	5.3% (51.1)	1.4% (4.8)	1.2% (12.9)	1.5% (40.7)
MSSES-Control	10.0% (6.1)	3.8% (30.0)	15.1% (47.2)	8.0% (8.4)	−0.2% (33.9)%	15.5% (68.3)	23.4% (9.5)	24.8%* (37.2)	22.1% (75.2)
IPA-Limitations	−4.1% (2.4)	−3.8% (18.6)	−4.6% (14.1)	−3.3% (2.6)	−6.3% (13.6)%	−0.7% (18.8)	−3.1% (2.8)	−5.3% (19.3)	−1.1% (15.9)
IPA-Problems	−3.4% (2.5)	−6.0% (17.0)	−1.7% (15.2)	−6.5% (2.9)	−8.4%* (18.35)	−4.9% (17.4)	−2.2% (3.3)	−3.9% (22.5)	−0.8% (18.1)

*, P < 0.01.

### HRQoL, anxiety, depression and fatigue

The mean (SD) MSQoL-54 (Physical, Mental), HADS (Anxiety, Depression) and MFIS-5 scores for the total group are presented in Table 4. The mean (SEM) percentage changes from baseline are shown in Table 5. In the total group the MSQoL-54 Mental and Physical scores had significantly increased at 6 months by 12.2% and 10.2%, respectively, compared to baseline, whereas the Mental scores were also significantly higher at 1 and 3 months (+16.0%, +13.3%) compared to baseline.

**Table 4:** Mean (SD) values of Multiple Sclerosis Quality of Life 54-Item (MSQoL-54), Hospital Anxiety and Depression Scale (HADS) and Modified Fatigue Impact 5-Item Scale (MFIS-5) scores at 1, 3 and 6 months after SCDP in the total group and in the Relapsing Remitting (RR) and Progressive (P) subgroups

	**Baseline**			**1 month**			**3 months**			**6 months**		
	**Total**	**RR**	**P**	**Total**	**RR**	**P**	**Total**	**RR**	**P**	**Total**	**RR**	**P**
MSQoL-54 physical	46.3 (13.1)	52.0 (13.1)	42.6 (11.6)	46.9 (15.4)	53.7 (14.6)	41.9 (14.2)	47.9 (18.4)	52.2 (16.7)	41.6 (16.9)	49.6 (16.6)	57.1 (15.2)	40.9 (11.6)
MSQoL-54 mental	52.8 (13.4)	51.2 (13.1)	54.1 (13.8)	59.6 (14.3)	60.5 (15.2)	58.8 (13.7)	59.0 (14.1)	59.8 (14.3)	57.5 (14.3)	57.3 (15.1)	60.6 (13.3)	53.6 (16.1)
HADS anxiety	7.67 (3.65)	6.78 (3.92)	8.35 (3.45)	6.59 (3.58)	5.39 (3.31)	7.41 (3.62)	6.56 (3.72)	5.67 (3.69)	7.45 (3.63)	6.53 (3.72)	5.11 (2.19)	7.81 (4.39)
HADS depression	6.38 (4.25)	6.00 (4.52)	6.61 (4.20)	5.76 (3.83)	4.67 (3.55)	6.55 (3.97)	5.61 (3.51)	5.11 (3.55)	6.18 (3.47)	5.95 (4.27)	4.72 (3.49)	7.14 (4.68)
MFIS-5	12.43 (3.65)	12.72 (3.16)	12.09 (4.08)	11.73 (3.43)	11.00 (3.31)	12.19 (3.53)	11.29 (3.78)	10.94 (3.59)	11.77 (3.95)	11.88 (3.49)	11.89 (3.55)	12.05 (3.50)

**Table 5:** Mean (SEM) percentage changes from baseline in Multiple Sclerosis Quality of Life 54-Item (MSQoL-54), Hospital Anxiety and Depression Scale (HADS) and Modified Fatigue Impact 5-Item Scale (MFIS-5) scores at 1, 3 and 6 months after SCDP in the total group and in the Relapsing Remitting (RR) and Progressive (P) subgroups

	**1 month**			**3 months**			**6 months**		
	**Total**	**RR**	**SP/PP**	**Total**	**RR**	**SP/PP**	**Total**	**RR**	**SP/PP**
MSQoL-54 physical	5.6% (4.7)	6.0% (5.0)	5.3% (8.2)	7.5% (6.2)	6.3% (5.9)	8.4% (10.7)	10.2%** (4.8)	12.7%** (5.5)	7.8% (8.0)
MSQoL-54 mental	16.0%* (5.0)	21.4%** (8.8)	11.5%** (5.4)	13.2%** (5.8)	22.3%*** (10.7)	5.5% (5.4)	12.2%** (6.0)	22.3%** (8.7)	3.2% (7.9)
HADS anxiety	−3.9% (12.6)	1.9% (27.2)	−9.9% (5.9)	−1.1% (13.7)	1.7% (27.7)	−3.5% (9.7)	−2.4% (10.1)	−2.6% (18.7)	−2.3% (9.9)
HADS depression	−3.4% (10.8)	−22.6%*** (11.0)	13.2% (17.8)	36.3% (24.8)	10.3% (19.6)	58.8% (43.1)	14.0% (15.6)	−10.6 (16.8)	36.3% (25.0)
MFIS-5	−2.2% (6.7)	−0.5% (12.4)	−4.0% (6.7)	−2.6 (7.5)	−2.3% (12.4)	−2.9% (9.0)	−0.33% (6.5)	3.0% (12.5)	−3.5% (4.7)

*, P < 0.005; **, P < 0.05; ***, 0.05 < P <0.06 (comparisons with baseline).

### RR and progressive groups

The mean (SD) values of MSSES-Function, MSSES-Control, IPA-Limitations and IPA-Problems scores in the RR and progressive subgroups at baseline and at 1, 3 and 6 months are presented in Table 2, and the mean (SEM) percentage changes from baseline at the various time points are shown in Table 3. Similarly, the corresponding values for Physical and Mental MSQoL-54, HADS Anxiety, HADS Depression, and MFIS-5 scores are given in Tables 4 and 5.

In the RR group the IPA-Problems score had significantly decreased (−8.4%) at 3 months and the MSSES-Control score had significantly increased (+24.8%) at 6 months, whereas no changes in primary outcomes were seen in the progressive group (Table 3). Moreover, in the RR group the mean MSQoL-54 Mental score had increased (+21.4%) at 1 month, and the Physical (+12.7%) and Mental (+22.3%) scores at 6 months, which was not the case in the progressive group (Table 5).

### Low- and high-disability groups

In patients with EDSS < 4.0 the mean IPA-Limitations and -Problems scores had significantly decreased (−6.1%, −8.8%) at 3 months, whereas the MSSES-Control score had increased (+23.8%) at 6 months (Table 6). In the EDSS > =4.0 group there were no significant changes. Moreover, at 1, 3 and 6 months the mean MSQoL-54 Mental score was higher than at baseline (+19.2%, +21.5%, +19.3%) in the EDSS < 4.0 group, in contrast to the higher EDSS group, where significant changes were absent (Table 7).

**Table 6:** Mean (SEM) percentage changes from baseline in Multiple Sclerosis Self-Efficacy Scale (MSSES) and Impact on Participation and Autonomy (IPA) scores at 1, 3 and 6 months after SCDP in PwMS with low disability (EDSS < 4.0) and high disability (EDSS > =4.0)

	**1 month**		**3 months**		**6 months**	
	**EDSS < 4.0**	**EDSS > =4.0**	**EDSS < 4.0**	**EDSS > =4.0**	**EDSS < 4.0**	**EDSS > =4.0**
MSSES-Function	3.2% (1.9)	5.4% (7.7)	−2.7% (5.1)	1.9% (9.6)	2.5% (3.0)	0.2% (9.5)
MSSES-Control	6.5% (6.5)	13.3% (10.4)	5.8% (6.5)	11.0% (16.6)	23.8%* (8.1)	22.9% (17.7)
IPA-Limitations	−6.3% (3.9)	−2.0% (3.0)	−6.1%** (3.0)	−0.6% (4.3)	−4.8% (4.0)	−1.3% (4.0)
IPA-Problems	−4.2% (4.2)	−2.9% (3.1)	−8.8%** (4.3)	−3.7% (4.0)	−1.3% (5.3)	−3.1% (4.1)

*, P < 0.01; **, 0.05 < P < 0.06 (comparisons with baseline).

**Table 7:** Mean (SEM) percentage changes from baseline in Multiple Sclerosis Quality of Life 54-Item (MSQoL-54), Hospital Anxiety and Depression Scale (HADS) and Modified Fatigue Impact 5-Item Scale (MFIS-5) scores at 1, 3 and 6 months after SCDP in PwMS with low disability (EDSS < 4.0) vs. high disability (EDSS > =4.0)

	**1 month**		**3 months**		**6 months**	
	**EDSS <4.0**	**EDSS > =4.0**	**EDSS <4.0**	**EDSS > =4.0**	**EDSS <4.0**	**EDSS > =4.0**
MSQoL-54 physical	7.7% (5.6)	3.4% (7.7)	9.0% (6.0)	5.9% (11.1)	9.7% (5.4)	10.7% (8.0)
MSQoL-54 mental	19.2%* (17.9)	12.7%** (6.1)	21.5%* (9.5)	4.4% (6.1)	19.3%* (8.3)	5.2% (8.6)
HADS anxiety	0.69% (25.9)	−5.9% (6.1)	4.8% (26.2)	−3.5% (10.3)	−1.6% (18.7)	−3.3% (8.5)
HADS depression	−12.7% (15.3)	8.6% (15.6)	17.4% (22.2)	57.5% (45.9)	6.9% (22.1)	21.1% (22.6)
MFIS-5	−3.0% (11.5)	−4.0% (7.1)	−4.5% (11.7)	−2.0% (9.9)	2.4% (11.9)	−3.2% (4.8)

*, P < 0.05; **, 0.05 < P <0.06 (comparisons with baseline).

## Discussion

In an observational study we assessed in PwMS the effect of a social cognitive wellness program with the participation of support partners on self-efficacy, participation and autonomy (primary outcomes), HRQoL, anxiety, depression and fatigue (secondary outcomes). In the total group a significant but moderate increase in mental HRQoL was seen at 1, 3 and 6 months, whereas improvements in the primary outcomes were absent. However, in the RR group the MSSE-Control score had significantly increased by 24.8% (mean) at 6 months, and the IPA-Problems score decreased by −8.4% (mean) at 3 months. Moreover, in RR patients the mental and physical HRQoL had increased at 6 months by 21.4% and 12.7% resp., with no such changes in the progressive group. These discrepancies between RR and progressive patients were mirrored by the results of the analyses according to degree of disability. Overall, our findings indicate that 3 to 6 months after SCDP, persons with RRMS or low disability may experience less limitations with respect to participation and autonomy, an improved confidence with managing symptoms and coping with demands of their disease (self-efficacy-control), and improved mental and physical HRQoL. Notably, the improved mental HRQoL (mean increase MSQoL-54 Mental score 22.3%) at 6 months is likely related to the improved self-efficacy-control (mean increase MSSES-Control score 24.8%), since in chronic conditions the relationship between self-efficacy and psychological well-being has been convincingly documented [6,3]. Moreover, the degree to which both these measures had increased (>20%) suggest that the observed changes are clinically relevant and do qualify as improvements.

Self-efficacy, being the belief in one’s ability to produce outcomes one wants [1,3], is a strong predictor of health behavior, and it can be instrumental in modulating the experience of chronic illness [18,3]. Various interventions have been developed to increase self-efficacy with the goal of improving chronic disease outcomes [3,19]. Some interventions have focused on a single behavior such as exercise or stress management, while others have taken a more comprehensive “lifestyle” approach [20,21]. In the U.S.A. a concentrated 4-day interdisciplinary educational wellness program has been developed for PwMS [8]. For conceptual and practical reasons we modified this program. We emphasized the social cognitive components and therefore only investigated couples of PwMS and their support partners. In addition, for reason of efficiency and to save time for the participants, notably the support partners, we condensed the program to 3 days by reducing the educational component. The beneficial changes after SCDP in the RR group are in line with the effects observed by Ng et al. after the 4-day original CDP [8]. These authors observed in MS patients (N = 98) improvements in self-efficacy control (MSSES-Control) and HRQoL (Short Form 36-Items [SF-36]) at 1, 3 and 6 months [8]; comparisons between RR and progressive PwMS were not reported [8]. Importantly and in contrast to our findings, improvements in the study by Ng et al. were independent from disability (EDSS). A comparison of their subjects’ disability level (median EDSS 3.5) with that in our RR (mean EDSS 3.1) and progressive (mean EDSS 5.5) groups, suggests that their participants were relatively mildly disabled and most likely mainly RR.

The negative findings in the progressive group may relate to several factors. The original CDP takes 4 days and consists of the following six components: group-based individual assessments (general health, gait, spirometry, visual acuity, muscle function, balance, coordination, fitness, exercise); group workshops (stretching, cognition, fatigue, Swiss ball, mindful motivation); group seminars/lectures (MS facts and treatment, MS research, sexual function, communication, psychological aspects of MS, nutrition, exercise, stress management, complementary and alternative medicine, goal setting); group optional activities (walking, stretching/yoga/Pilates); individual consultations (neurological, psychological, program summary, goal setting); and individual optional consultations (program integration and skill building, speech language pathologist, occupational therapist, aqua therapist, nurse practitioner, physiatrist, dietician, urologist) [8]. It may be that the social cognitive approach, the reduced educational component, or the condensation to 3 days have rendered our SCDP less effective in persons with progressive MS. On the other hand, the relatively small numbers of participants in our study may have prevented the detection of small beneficial effects.

Depression and anxiety are common in PwMS. People’s mood can be significantly affected by the perception of their disability [22], and in a recent cohort study in PwMS it was shown that experiencing depression significantly predicted anxiety [22]. In our study we only observed in the RR group a tendency to a temporary decrease in the depression score (−22.6%) at 1 month. Changes on the longer term were not seen. Neither was the level of fatigue (MFIS-5) affected by SCDP.

With greater loss of mobility, the ability to perform activities of daily living decreases and the dependence upon the assistance of others increases [23,24]. In the RR group the MS-related limitations, and the extent to which these limitations were experienced as problematic, were decreased at 3 months, but not at 6 months. This finding suggests that the effect of a single SCDP intervention may be temporary, and that follow-up sessions should be considered.

Given the uncontrolled design of the study we cannot infer that SCDP caused the changes that we observed. Yet, a placebo effect and a regression to the mean seem insufficient explanations for the improvements at 3 or 6 months, as virtually no changes were seen in persons with progressive MS. Another limitation of our study is the relatively small number of PwMS studied.

## Conclusions

Our findings suggest that in PwMS with a RR disease course or low disability, an intense multi- and interdisciplinary, 3-day, social cognitive wellness program with the participation of support partners, may lead to improvements in self-efficacy-control, and mental and physical HRQoL 6 months later. The data also suggest that social cognitive wellness programs involving support partners may enhance autonomy and participation. To be optimally effective, social cognitive wellness programs for persons with a chronic disease may have to be differentiated according to type of disease or degree of disability. Actually we conduct a randomised controlled trial on the effectiveness - in terms of self-efficacy, autonomy and participation, and HRQoL - of SCDP in persons with RRMS and low disability (EDSS < 4.0). Its primary endpoint, the mean of the MSSES-Control scores at 3 and 6 months, is based on the observational data from the present study.

## Competing interests

The study was funded by the National Multiple Sclerosis Foundation (NMSF), Maassluis, the Netherlands, including the article-processing charge. Peter Joseph Jongen has received honoraria from Allergan, Almirall, Biogen-Idec, Merck-Serono, Novartis, sanofi-aventis, and Teva for contributions to symposia as a speaker, consultancy activities or investigator-initiated and –driven research projects.

## Authors’ contributions

PJJ conceived and designed the study, coordinated the acquisition of data, analyzed the data, interpreted the data, drafted the manuscript, and has given final approval of the version to be published. RR conceived and developed the SCDP, was a member of the multidisciplinary team, has been involved in the analysis and interpretation of data, has revised the manuscript critically for important intellectual content, and has given final approval of the version to be published. MH was involved in the development of SCDP, was a member of the multidisciplinary team, has been involved in the acquisition and interpretation of data, has revised the manuscript critically for important intellectual content, and has given final approval of the version to be published. AH was involved in the development of the SCDP, in the organization of the study, and in the acquisition of data, was a member of the multidisciplinary team, has revised the manuscript critically for important intellectual content, and has given final approval of the version to be published. LD was involved in the development of SCDP, was a member of the multidisciplinary team, has been involved in the acquisition of data, has revised the manuscript critically for important intellectual content, and has given final approval of the version to be published. AvdZ conceived and developed the SCDP, has been involved in the analysis and interpretation of data, has revised the manuscript critically for important intellectual content, and has given final approval of the version to be published. HW was involved in the development of SCDP, was a member of the multidisciplinary team, has been involved in the acquisition of data, has revised the manuscript critically for important intellectual content, and has given final approval of the version to be published. JV was involved in the development of SCDP, was a member of the multidisciplinary team, has been involved in the acquisition of data, has revised the manuscript critically for important intellectual content, and has given final approval of the version to be published. HW was involved in the development of SCDP, was a member of the multidisciplinary team, has been involved in the acquisition of data, has revised the manuscript critically for important intellectual content, and has given final approval of the version to be published. MvD was involved in the development of SCDP, organized the SCDP, has been involved in the acquisition of data, has revised the manuscript critically for important intellectual content, and has given final approval of the version to be published. WL and RD have been involved in the analysis and interpretation of data, have revised the manuscript critically for important intellectual content, and have given final approval of the version to be published. LHV was involved in the development of SCDP, was a member of the multidisciplinary team, has been involved in the acquisition and interpretation of data, has revised the manuscript critically for important intellectual content, and has given final approval of the version to be published. All authors read and approved the final manuscript.

## Authors’ information

PJJ was co-founder (1996), and director and senior neurologist (1996–2007) of the Multiple Sclerosis Centre Nijmegen, Nijmegen, the Netherlands. In 2008 he founded the MS4 Research Institute, an independent, non-profit, organization for patient-centered research in the field of MS. PJJ is member of the Scientific Medical Board of the Multiple Sclerosis International Federation (MSIF), former member of the Subcommittee on Multiple Sclerosis/Demyelinative Diseases of the European Neurological Society (ENS) and former council member of the European Committee on Treatment and Research in Multiple Sclerosis (ECTRIMS). He is (co-)author of more than 95 scientific papers in peer-reviewed journals.
